# Intensity of Leisure-Time Exercise and Risk of Depressive Symptoms Among Japanese Workers: A Cohort Study

**DOI:** 10.2188/jea.JE20170009

**Published:** 2018-02-05

**Authors:** Keisuke Kuwahara, Toru Honda, Tohru Nakagawa, Shuichiro Yamamoto, Takeshi Hayashi, Tetsuya Mizoue

**Affiliations:** 1 Department of Epidemiology and Prevention, Bureau of International Health Cooperation, National Center for Global Health and Medicine, Tokyo, Japan; 2 Teikyo University Graduate School of Public Health, Tokyo, Japan; 3 Hitachi, Ltd., Ibaraki, Japan

**Keywords:** intensity, physical activity, cohort studies, depressive symptoms, prevention

## Abstract

**Background:**

Data on the effect of physical activity intensity on depression is scarce. We investigated the prospective association between intensity of leisure-time exercise and risk of depressive symptoms among Japanese workers.

**Methods:**

The participants were 29,052 employees (24,653 men and 4,399 women) aged 20 to 64 years without psychiatric disease including depressive symptoms at health checkup in 2006–2007 and were followed up until 2014–2015. Details of leisure-time exercise were ascertained via a questionnaire. Depressive states were assessed using a 13-item questionnaire. Multivariable-adjusted hazard ratio of depressive symptoms was estimated using Cox regression analysis.

**Results:**

During a mean follow-up of 5.8 years with 168,203 person-years, 6,847 workers developed depressive symptoms. Compared with workers who engaged in no exercise during leisure-time (0 MET-hours per week), hazard ratios (95% confidence intervals) associated with >0 to <7.5, 7.5 to <15.0, and ≥15.0 MET-hours of leisure-time exercise were 0.88 (0.82–0.94), 0.85 (0.76–0.94), and 0.78 (0.68–0.88) among workers who engaged in moderate-intensity exercise alone; 0.93 (0.82–1.06), 0.82 (0.68–0.98), and 0.83 (0.71–0.98) among workers who engaged in vigorous-intensity exercise alone; and 0.96 (0.80–1.15), 0.80 (0.67–0.95), and 0.76 (0.66–0.87) among workers who engaged in both moderate- and vigorous-intensity exercise with adjustment for age, sex, lifestyles, work-related and socioeconomic factors, and body mass index. Additional adjustment for baseline depression score attenuated the inverse association, especially among those who engaged in moderate-intensity exercise alone.

**Conclusions:**

The results suggest that vigorous-intensity exercise alone or vigorous-intensity combined with moderate-intensity exercise would prevent depressive symptoms among Japanese workers.

## BACKGROUND

Depression is a major concern in the world,^1^ and physical activity is a promising non-pharmaceutical intervention for preventing depression. A meta-analysis reported that physical activity intervention reduced depressive symptoms in adults without clinical depression.^2^ Although physical activity guidelines equally recommend at least 150 minutes of moderate-intensity activity per week, or 75 minutes of vigorous-intensity activity, or an equivalent volume (expressed as duration of time engaged in the activity multiplied by its intensity) of the combined intensities for health, including mental health,^3^ evidence is limited on the effect of different intensities of physical activity on mental health.

A few cohort studies^4^^–^^6^ have examined the association of intensity of physical activity with risk of depressive symptoms or depression; except one study,^4^ all^5^^,^^6^ showed a greater decrease in the risk with increasing exercise intensity. However, the earliest study of men^4^ adjusted only for age, and confounding may have affected their results. Further, the risk reduction observed in one of the three studies may be ascribed to greater volume (eg, expressed as metabolic equivalent [MET]-hours) of the greater intensity of the activity.^5^ To minimize such a possibility, the volume of physical activity should be controlled in the analysis.^7^ In another study,^6^ where the volume (MET-min per week) was considered, the risk associated with vigorous activity alone was not examined, as individuals who engaged in such activity alone were few.

We recently reported a U-shaped association between volume of leisure-time exercise and risk of depressive symptoms among Japanese individuals.^8^ Here, we compared the risk of depressive symptoms by exercise intensity with consideration of exercise volume, defined as MET-hours among Japanese workers.

## METHODS

### Study design

The present study is based on a sub-cohort of the Japan Epidemiology Collaboration on Occupational Health (J-ECOH) Study, an on-going, large-scale, multi-company study of Japanese workers, as described elsewhere.^9^^,^^10^ In Japan, workers are obliged to undergo health checkups annually. Before data collection, the conduct of the J-ECOH Study was announced in each of the participating companies using posters to explain the purpose and procedures of the study. Participants did not provide verbal or written informed consent to take part in the study, but they were given the opportunity to refuse participation. This procedure follows the Japanese Ethical Guidelines for Epidemiological Research. The study protocol was approved by the Ethics Committee of the National Center for Global Health and Medicine, Japan. Participants were 50,246 workers (41,039 men and 9,207 women) aged 20 to 64 years who underwent health examinations from April 2006 through March 2007 (baseline) at one of the participating companies, where detailed data on physical activity have been available. We followed the participants up through March 2015 using health checkup data.

### Participants

Of 50,246 workers, 16,412 were excluded at baseline for the following reasons: missing data regarding baseline depression score; presence of depressive symptoms (depression score of ≥26 points); or a history of psychiatric disease, cancer, ischemic heart disease, or stroke. We additionally excluded workers who had any missing data on leisure-time exercise (*n* = 1,485) or covariates (*n* = 1,873). Lastly, we excluded 1,424 workers who did not attend any subsequent health examination or did not complete questions on mental health after baseline examination, leaving 29,052 workers (24,653 men and 4,399 women) aged 20 to 64 years (mean age: 42.7 years) for analysis.

### Intensity and volume of leisure-time exercise

Assessment of leisure-time exercise volume and intensity has been described elsewhere.^9^ Briefly, MET of the activity was estimated using the standard physical activity compendium. Weekly volume (MET-hours) of leisure-time exercise was calculated using data on type, frequency, and duration of up to three activities during leisure. Then, participants were divided into 10 groups according to the combination of exercise volume (none, >0 to <7.5, 7.5 to 15.0, and ≥15.0 MET-hours per week) and intensity (moderate alone [3 to 6 MET], vigorous alone [>6 MET], and both).

### Depressive symptoms

Depressive symptoms were assessed using a questionnaire composed of 13 questions on subjective symptoms related to depression.^11^ This questionnaire used phrases similar to that in commonly-used questionnaires for depressive symptoms, including Center for Epidemiologic Studies Depression Scale^12^ and Self-rating Depression Scale (SDS).^13^ The total depression score (13 to 52 points) was calculated as the sum of the scores across the questions if the participant completed all 13 questions. This score is highly correlated with the SDS score (*r* = 0.75).^11^ Because there is no standard cutoff of the score to identify depressive symptoms, we defined the cutoff for diagnosis of depressive symptoms as 26 points or more (top 25% of scores) based on the prevalence of depressive symptoms among Japanese workers.^14^^,^^15^ We considered incident cases of depressive symptoms as those who met the criteria of depressive symptoms at the follow-up examinations after baseline. For participants who did not develop depressive symptoms, we used the last examination during follow-up as the censor date.

### Covariates

Body height and weight were measured to the nearest 0.1 cm and 0.1 kg, respectively. Body mass index (BMI) was determined as weight (kg) divided by squared height (m^2^). We obtained data on history of disease, smoking, alcohol use, sleep, shift work and overtime work, occupational physical activity, time spent walking to and from work, job position, and marital status using a standard questionnaire.^8^

### Statistical analysis

Data are shown as mean (standard deviation) for continuous variables and number (percentages) for categorical variables. Hazard ratios (HRs) and their 95% confidence intervals (CIs) for depressive symptoms were calculated using Cox proportional regression analysis. Age (years, continuous) and sex were adjusted for in model 1. In model 2, smoking (non-smokers, smokers consuming 1 to 10, 11 to 20, or ≥21 cigarettes per day), alcohol consumption (non-drinkers, drinkers consuming <1, 1 to <2, or ≥2 *go* of Japanese sake equivalent per day; 1 *go* of Japanese sake contains about 23 g of ethanol), sleep duration (<5, 5 to 6, 6 to <7, or ≥7 hours per day), time spent walking to and from work (<20, 20 to <40, or ≥40 min per day), occupational physical activity (mostly sedentary, mostly standing or walking, or fairly physically active), shift work (yes or no), monthly duration of overtime work (<45, 45 to 60, or ≥60 hours), job position (high or low), marital status (married or not), and body mass index (<18.5, 18.5 to <23.0, 23.0 to <25.0, 25.0 to <30.0, or ≥30.0 kg/m^2^) were additionally adjusted for. In model 3, baseline depression score (continuous) was further adjusted for. The variance inflation factor, an indicator of multicollinearity, in each variable was less than 1.5 in the fully adjusted model. For the sensitivity analysis, we repeated the main analysis after exclusion of participants with a short follow-up duration (<3 years). Two-sided *P* values <0.05 were considered statistically significant. All analyses were performed using Stata 14.1 (StataCorp, College Station, TX, USA).

## RESULTS

Baseline characteristics of participants according to combinations of leisure-time exercise volume and intensity are shown in Table 1. Individuals who did not engage in exercise tended to be female, slightly older, married, and more depressed than those who engaged in exercise. They also tended to be smokers, heavy drinkers, and short sleepers.

**Table 1.** Baseline characteristics of participants by intensity and volume of leisure-time exercise

	None	Low volume			Medium volume			High volume		
		
		MPA alone	VPA alone	Both	MPA alone	VPA alone	Both	MPA alone	VPA alone	Both
Number of participants	17,690	4,113	796	456	1943	489	631	1,331	596	1,007
Men	14,597 (82.5)	3,582 (87.1)	688 (86.4)	394 (86.4)	1,754 (90.3)	422 (86.3)	552 (87.5)	1,229 (92.3)	517 (86.7)	918 (91.2)
Age, years	43.1 (10.2)	42.5 (10.8)	35.9 (9.5)	40.6 (10.1)	44.2 (10.7)	37.3 (10.3)	41.2 (11.1)	45.4 (11.2)	39.2 (11.3)	42.4 (11.5)
BMI, kg/m^2^	23.3 (3.4)	23.5 (3.4)	22.9 (3.1)	23.1 (3.0)	23.8 (3.2)	22.9 (3.2)	23.2 (3.0)	24.0 (3.2)	22.7 (2.8)	23.3 (2.8)
Depression score	18.7 (3.7)	18.2 (3.7)	18.9 (3.7)	18.3 (3.8)	17.9 (3.7)	18.7 (3.6)	18.1 (3.8)	17.5 (3.6)	18.5 (3.7)	17.8 (3.6)
Smoking	7,660 (43.3)	1,723 (41.9)	320 (40.2)	157 (34.4)	853 (43.9)	167 (34.2)	185 (29.3)	602 (45.2)	169 (28.4)	318 (31.6)
Heavy drinking^a^	1,517 (8.6)	320 (7.8)	26 (3.3)	32 (7.0)	183 (9.4)	21 (4.3)	38 (6.0)	129 (9.7)	44 (7.4)	83 (8.2)
Sleeping, <6 hours	8,682 (49.1)	1,817 (44.2)	389 (48.9)	213 (46.7)	832 (42.8)	252 (51.5)	299 (47.4)	544 (40.9)	265 (44.5)	449 (44.6)
Low job position	14,719 (83.2)	3,403 (82.7)	722 (90.7)	374 (82.0)	1,512 (77.8)	416 (85.1)	499 (79.1)	1,051 (79.0)	504 (84.6)	798 (79.3)
Shift work	3,385 (19.1)	810 (19.7)	163 (20.5)	75 (16.5)	337 (17.3)	74 (15.1)	96 (15.2)	243 (18.3)	90 (15.1)	147 (14.6)
Long overtime work^b^	5,733 (32.4)	1,253 (30.5)	264 (33.2)	147 (32.3)	584 (30.1)	174 (35.6)	210 (33.3)	332 (24.9)	201 (33.7)	295 (29.3)
Sedentary work	10,280 (58.1)	2,384 (58.0)	481 (60.4)	284 (62.3)	1,194 (61.5)	333 (68.1)	416 (65.9)	741 (55.7)	377 (63.3)	630 (62.6)
Low CA^c^	9,482 (53.6)	2,282 (55.5)	482 (60.6)	234 (51.3)	1,055 (54.3)	281 (57.5)	337 (53.4)	782 (58.8)	316 (53.0)	566 (56.2)
Unmarried	4,963 (28.1)	1,171 (28.5)	324 (40.7)	155 (34.0)	489 (25.2)	201 (41.1)	210 (33.3)	308 (23.1)	221 (37.1)	326 (32.4)

BMI, body mass index; CA, commuting activity; MPA, moderate physical activity; VPA, vigorous physical activity.

Data are shown as mean (standard deviation) for continuous variables and number (percentages) for categorical variables. Participants were divided by exercise volume (0, >0 to <7.5, 7.5 to <15.0, and ≥15.0 MET-hours per week) and types of intensity engaged (moderate-intensity exercise alone, vigorous-intensity exercise alone, and both intensities).

^a^Consuming 2 *go* or more of Japanese sake equivalent per day.

^b^≥45 hours of overtime work per month.

^c^<20 min of walking to and from work per day.

**Figure 1.**
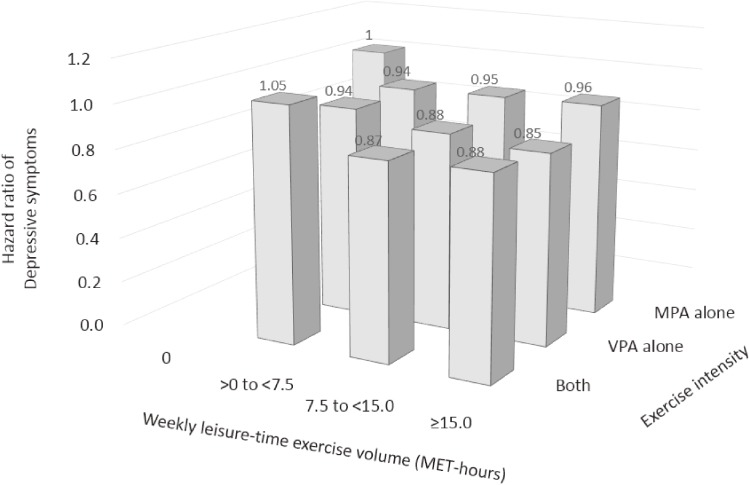
Hazard ratio of developing depressive symptoms according to the volume and intensity of leisure-time exercise. Data were adjusted for age (continuous, year), sex, smoking (non-smokers, smokers consuming 1 to 10, 11 to 20, or ≥21 cigarettes per day), alcohol consumption (non-drinkers, drinkers consuming <1, 1 to <2, or ≥2 go of Japanese sake equivalent per day), sleep duration (<5, 5 to <6, 6 to <7, or ≥7 hours per day), walking to and from work (<20, 20 to <40, or ≥40 min per day), occupational physical activity (sedentary, mostly standing or walking, or fairly physically active), shift work (yes or no), monthly overtime work (<45, 45 to 60, 60 to <80, 80 to <100, or ≥100 hours), job position (high or low), marital status (married or not), body mass index (<18.5, 18.5 to <23.0, 23.0 to <25.0, 25.0 to <30.0, or ≥30.0 kg/m^2^), and depression score (continuous) at baseline. No exercise was treated as reference. MPA, moderate-intensity physical activity; VPA, vigorous-intensity physical activity.

During a mean follow-up duration of 5.8 years, with 168,203 person-years, 6,847 workers newly developed depressive symptoms. Table 2 shows the risk of depressive symptoms according to the combinations of leisure-time exercise volume and intensity. Risk reduction was comparable across the different exercise intensities after adjustment for potential confounders except the baseline depression score (model 2). For example, compared with no weekly exercise (0 MET-hours), the HRs of depressive symptoms associated with ≥15.0 MET-hours were 0.78 (95% CI, 0.68–0.88) among workers who engaged in moderate-intensity exercise alone, 0.83 (95% CI, 0.71–0.98) among workers who engaged in vigorous-intensity exercise alone, and 0.76 (95% CI, 0.66–0.87) among workers who engaged in vigorous-intensity combined with moderate-intensity exercise, respectively. Additional adjustment for baseline depression score attenuated the risk reduction, especially for moderate-intensity exercise alone, and all the risk reductions became non-significant (model 3), as shown in Figure 1. The HRs of depressive symptoms associated with ≥15.0 MET-hours were 0.96 (95% CI, 0.84–1.09) for moderate-intensity exercise alone; 0.85 (95% CI, 0.72–1.00; *P* = 0.05) for vigorous-intensity exercise alone; and 0.88 (95% CI, 0.77–1.02) for vigorous-intensity combined with moderate-intensity exercise, respectively. In a sensitivity analysis, the exclusion of participants with short-term follow-up gave similar results (data not shown).

**Table 2.** Association between risk of depressive symptoms and combinations of leisure-time exercise intensity and volume among 29 112 Japanese workers

	Inactive		Low volume		Physical activity meeting recommendation			
		
	Cases (*n*)/ Person-years	HR	Cases (*n*)/ Person-years	HR (95% CI)	Median volume		High volume	
	
					Cases (*n*)/ Person-years	HR (95% CI)	Cases (*n*)/ Person-years	HR (95% CI)
Model 1^a^								
	4,321/101,082	1						
MPA alone			920/24,260	0.86 (0.80, 0.93)	399/11,512	0.84 (0.75, 0.93)	238/7,794	0.76 (0.67, 0.86)
VPA alone			241/4,769	0.93 (0.81, 1.06)	120/2,846	0.83 (0.70, 0.99)	150/3,624	0.82 (0.70, 0.97)
Both			120/2,705	0.95 (0.80,1.14)	136/3,680	0.80 (0.67, 0.95)	198/5,930	0.75 (0.65, 0.87)
Model 2^b^								
		1						
MPA alone				0.88 (0.82, 0.94)		0.85 (0.76, 0.94)		0.78 (0.68, 0.88)
VPA alone				0.93 (0.82, 1.06)		0.82 (0.68, 0.98)		0.83 (0.71, 0.98)
Both				0.96 (0.80, 1.15)		0.80 (0.67, 0.95)		0.76 (0.66, 0.87)

CI, confidence interval; HR, hazard ratio; MPA, moderate-intensity physical activity; VPA, vigorous-intensity physical activity.

^a^Adjusted for age (continuous, year) and sex.

^b^Additionally adjusted for smoking (non-smokers, smokers consuming 1 to 10, 11 to 20, or ≥21 cigarettes per day), alcohol consumption (non-drinkers, drinkers consuming <1, 1 to <2, or ≥2 *go* of Japanese sake equivalent per day), sleep duration (<5, 5 to <6, 6 to <7, or ≥7 hours per day), walking to and from work (<20, 20 to <40, or ≥40 min per day), occupational physical activity (sedentary, mostly standing or walking, or fairly physically active), shift work (yes or no), monthly overtime work (<45, 45 to 60, 60 to <80, 80 to <100, or ≥100 hours), job position (high or low), marital status (married or not), and body mass index (<18.5, 18.5 to <23.0, 23.0 to <25.0, 25.0 to <30.0, or ≥30.0 kg/m^2^).

## DISCUSSION

The present study showed that, when compared with no exercise, vigorous-intensity exercise alone was associated with a 15% lower risk of developing depressive symptoms, and vigorous-intensity combined with moderate-intensity exercise was associated with a 12% lower risk, whereas moderate-intensity exercise alone was not associated with depressive symptoms, after adjustment for baseline depressive state. However, these reductions did not reach statistical significance. This is one of the few studies addressing the association between the intensity of physical activity and risk of depressive symptoms.

We found greater reduction in the risk of depressive symptoms among those who performed vigorous-intensity exercise than among those who performed moderate-intensity exercise; this finding is consistent with the results of some previous studies^5^^,^^6^ but contrasts with the results of other^4^ cohort studies on depression or depressive symptoms. Of note, three studies compared the risks between light-intensity and moderate- to vigorous-intensity exercise,^5^ moderate-intensity exercise alone and moderate- to vigorous-intensity exercise^6^ or light exercise only, light to moderate exercise, and moderate-only sports activities.^4^ Thus, the association of vigorous-intensity activity alone has been unaddressed; the present study fills this gap. However, caution is needed to interpret the present results because the risk reductions were statistically non-significant, even at the highest volume of activity (*P* < 0.1). Thus, although vigorous-intensity exercise may be more beneficial than moderate exercise for the prevention of depression, we cannot conclude definitely.

The mechanisms involved in the greater reduction in the risk of depressive symptoms with greater intensity of activity are not fully understood. An intervention study in healthy young men showed that vigorous-intensity exercise, compared with moderate-intensity, resulted in more frequent large increases in levels of brain-derived neurotrophic factor,^16^ a key factor for the regulation of mood.^17^ Additionally, greater exercise intensity has been shown to decrease more visceral fat than lesser intensity,^18^ which increases inflammation,^19^ an important pathophysiologic factor in depression.^20^ A recent cross-sectional study reported a positive association between visceral fat area and depressive symptoms.^21^

The strengths of this study include the large sample size and annual assessment of depressive symptoms during follow-up. In addition, we could minimize the confounding by physical activity volume on the association of physical activity intensity and depression by comparing the risks at the same range of exercise volume. Further, baseline depressive state may be associated with physical activity level and is a predictor of the development of depressive symptoms, so baseline depressive state may confound the association of physical activity with future depression. However, previous studies did not adjust for the baseline depressive state.^4^^–^^6^ In the present study, after adjustment for baseline depression score, risk reduction was evident in those who performed vigorous-intensity exercise alone and vigorous-intensity combined with moderate-intensity exercise.

Some limitations of this study should also be noted. First, the validity and reliability of the present questionnaire on physical activity are unclear. However, the activity questionnaire is similar to validated and reproducible questionnaires on physical activity.^22^^,^^23^ Additionally, the present physical questionnaire assessed exercise or sports activities of three METs or more, thus, we could not examine influence of the light-intensity activity. Second, although the present questionnaire for depressive symptoms has good internal consistency and good concurrent validity with SDS,^11^ it was not validated against clinically diagnosed depression. Further, construct validity of the present depression questionnaire, which are shown by relationship with related phenomenon, including social support, self-rated health, or stressful life events,^24^ is unclear. Third, we do not have data on the accuracy of the depression questionnaire. However, given the high correlation and good agreement between the present questionnaire and SDS,^11^ the accuracy may not affect the main findings. Fourth, there is no standard cutoff for the present questionnaire on depressive symptoms, so we defined the cutoff to correspond to the prevalence of depressive symptoms among Japanese workers (20–30%).^14^^,^^15^ Fifth, although we adjusted for many potential confounders, unmeasured factors, including income and caring responsibilities for child or elderly, may have affected the results. Nonetheless, when we repeated the analysis after exclusion of women, who might have different caring responsibilities as compared with men, the results were not largely changed (data not shown). Lastly, the participants were predominantly male workers in a large-scale electrical and machinery and apparatus manufacturing company. Therefore, the present findings may not be applicable to female workers, workers in companies with different backgrounds, the general working population, the elderly, or the unemployed.

In summary, results of the present study among Japanese workers indicate that vigorous-intensity exercise but not moderate-intensity exercise may be associated with a lower risk of depressive symptoms, although the risk reductions did not reach statistical significance. Larger cohort studies are needed to confirm the present findings.
